# The Expanding Functions of Cellular Helicases: The Tombusvirus RNA Replication Enhancer Co-opts the Plant eIF4AIII-Like AtRH2 and the DDX5-Like AtRH5 DEAD-Box RNA Helicases to Promote Viral Asymmetric RNA Replication

**DOI:** 10.1371/journal.ppat.1004051

**Published:** 2014-04-17

**Authors:** Nikolay Kovalev, Peter D. Nagy

**Affiliations:** Department of Plant Pathology, University of Kentucky, Lexington, Kentucky, United States of America; Harvard Medical School, United States of America

## Abstract

Replication of plus-strand RNA viruses depends on recruited host factors that aid several critical steps during replication. Several of the co-opted host factors bind to the viral RNA, which plays multiple roles, including mRNA function, as an assembly platform for the viral replicase (VRC), template for RNA synthesis, and encapsidation during infection. It is likely that remodeling of the viral RNAs and RNA-protein complexes during the switch from one step to another requires RNA helicases. In this paper, we have discovered a second group of cellular RNA helicases, including the eIF4AIII-like yeast Fal1p and the DDX5-like Dbp3p and the orthologous plant AtRH2 and AtRH5 DEAD box helicases, which are co-opted by tombusviruses. Unlike the previously characterized DDX3-like AtRH20/Ded1p helicases that bind to the 3′ terminal promoter region in the viral minus-strand (−)RNA, the other class of eIF4AIII-like RNA helicases bind to a different *cis*-acting element, namely the 5′ proximal RIII(−) replication enhancer (REN) element in the TBSV (−)RNA. We show that the binding of AtRH2 and AtRH5 helicases to the TBSV (−)RNA could unwind the dsRNA structure within the RIII(−) REN. This unique characteristic allows the eIF4AIII-like helicases to perform novel pro-viral functions involving the RIII(−) REN in stimulation of plus-strand (+)RNA synthesis. We also show that AtRH2 and AtRH5 helicases are components of the tombusvirus VRCs based on co-purification experiments. We propose that eIF4AIII-like helicases destabilize dsRNA replication intermediate within the RIII(−) REN that promotes bringing the 5′ and 3′ terminal (−)RNA sequences in close vicinity via long-range RNA-RNA base pairing. This newly formed RNA structure promoted by eIF4AIII helicase together with AtRH20 helicase might facilitate the recycling of the viral replicases for multiple rounds of (+)-strand synthesis, thus resulting in asymmetrical viral replication.

## Author Summary

Genome-wide screens for host factors affecting tombusvirus replication in yeast indicated that subverted cellular RNA helicases likely play major roles in virus replication. Tombusviruses do not code for their own helicases and they might recruit host RNA helicases to aid their replication in infected cells. Accordingly, in this paper, the authors show that the yeast eIF4AIII-like Fal1p and Dbp3p and the orthologous plant AtRH2 and AtRH5 DEAD-box helicases are co-opted by *Tomato bushy stunt virus* (TBSV) to aid viral replication. The authors find that eIF4AIII-like helicases bind to the replication enhancer element (REN) in the viral (−)RNA and they promote (+)-strand TBSV RNA synthesis *in vitro*. Data show that eIF4AIII-like helicases are present in the viral replicase complex and they bind to the replication proteins. In addition, the authors show synergistic effect between eIF4AIII-like helicases and the previously identified DDX3-like Ded1p/AtRH20 DEAD box helicases, which bind to a different *cis*-acting region in the viral (−)RNA, on stimulation of plus-strand synthesis. In summary, the authors find that two different groups of cellular helicases promote TBSV replication via selectively enhancing (+)-strand synthesis through different mechanisms.

## Introduction

Host factors co-opted for replication of plus-stranded (+)RNA viruses are critical in each step of the well-orchestrated infection process. After translation of the viral mRNA-sense genomic RNA(s), the viral (+)RNA and the viral replication proteins together with host RNA-binding proteins (RBPs) are recruited to the site of viral replication in membranous cellular compartments. Ultimately, the process leads to the assembly of the membrane-bound viral replicase complexes (VRCs), followed by the activation of the polymerase function of the viral RNA-dependent RNA polymerase (RdRp), and initiation of complementary RNA synthesis on the viral (+)RNA template [1]–[4]. Subsequent (+)-strand synthesis in the VRCs takes place in an asymmetric manner, producing excess amounts of (+)-strand progeny, which is released from replication to participate in encapsidation, cell-to-cell movement and other viral processes.

Although the roles of host factors in facilitating the replication process of (+)RNA viruses have been extensively characterized in recent years [1]–[3], [5]–[11], our current understanding of the role of cellular RBPs, which constitute one of the largest groups of host factors identified is incomplete [1], [12], [13]. The co-opted RBPs likely affect several steps in viral RNA replication, including viral (+)RNA recruitment, stabilization of the viral RNA, VRC assembly and viral RNA synthesis.

*Tomato bushy stunt virus* (TBSV) is a plant RNA virus with a single ∼4,800 nt genomic RNA and has two essential replication proteins, p33 and p92^pol^, required for TBSV replicon (rep)RNA replication in yeast (*Saccharomyces cerevisiae*) model host [14], [15]. The membrane-bound tombusvirus VRC contains p33 and p92^pol^, and the tombusviral (+)repRNA, which serves both as a template and as a platform during VRC assembly and activation [16]–[20]. Interestingly, the tombusvirus VRC contains at least seven host proteins as resident members, including glyceraldehyde-3-phosphate dehydrogenase (GAPDH, encoded by *TDH2* and *TDH3* in yeast) [21], the heat shock protein 70 chaperones (Hsp70, Ssa1/2p in yeast) [22]–[25], pyruvate decarboxylase (Pdc1p) [25], Cdc34p E2 ubiquitin conjugating enzyme [26], eukaryotic translation elongation factor 1A (eEF1A) [27], [28], eEF1Bγ [29], and Ded1p DEAD-box helicase [30]. The VRC also contains two temporary resident proteins, Pex19p shuttle protein [31] and the Vps23p adaptor ESCRT protein [28], [32], [33]. Detailed mechanistic studies revealed that the cellular Hsp70, eEF1A and Vps23p are involved in the assembly of the VRC, while the functions of host RBPs, such as eEF1A, eEF1Bγ, GAPDH and Ded1p, are to regulate viral RNA synthesis by the VRC [3], [21]–[24], [29], [30], [34].

In spite of our growing understanding of TBSV replication and TBSV-host interaction, many questions remain. Indeed, multiple genome-wide screens and global proteomics approaches with TBSV using yeast as host identified ∼500 host factors, which interact with viral replication proteins or affect TBSV replication [25], [26], [28], [34]–[38]. Among the host proteins identified are 11 host ATP-dependent RNA helicases out of 39 known yeast helicases that could be involved in TBSV replication. DEAD-box proteins constitute the largest family of RNA helicases, which perform ATP-dependent RNA duplex unwinding, RNA folding, remodeling of RNA-protein complexes, and RNA clamping in cells. DEAD-box helicases are involved in all aspects of cellular metabolism [39]–[41] and affect replication of many viruses [42], including plant RNA viruses [43]. Plant RNA helicases are also implicated in plant responses to abiotic stress and pathogen infections [44]–[46].

The many cellular RNA helicases identified in TBSV screens are intriguing because, tombusviruses and other small RNA viruses do not code for their own helicases [47], [48]. These viruses likely recruit host helicases in order to facilitate viral replication. The best-characterized member of the helicase family involved in tombusvirus replication is the yeast Ded1p (the human DDX3-like) and the similar plant AtRH20 DEAD-box helicases, both of which promote (+)-strand synthesis [30]. Ded1p and AtRH20 bind to the 3′-end of the TBSV minus-strand RNA, making the promoter sequence accessible to p92^pol^ for initiation of (+)-strand RNA synthesis. Additional characterization of Ded1p and the similar yeast Dbp2p (similar to human p68) DEAD-box helicases revealed that these helicases play major and overlapping roles in (+)-strand synthesis [30], [49]. Altogether, the identification of 11 yeast RNA helicases involved in tombusvirus replication suggests that tombusviruses likely co-opt a number of host RNA helicases and these helicases might have a number of unique functions in viral replication.

In this paper, we characterized the novel pro-viral functions of two yeast RNA helicases, which were among those identified in previous screens, and their plant orthologs in TBSV replication. We found that the yeast Dbp3p (DEAD box protein 3, human DDX5-like) and Fal1p (eukaryotic translation initiation factor 4AIII-like) DEAD box helicases, which are involved in ribosome biogenesis in yeast [50]–[52], and the orthologous *Arabidopsis* RH2 and RH5 helicases bind to a critical replication enhancer element (REN) present in a 5′ proximal region of the TBSV minus-strand RNA. We show that these cellular helicases can locally unwind the double-stranded (ds) structure within the REN of the replication intermediate *in vitro*. These activities by the host helicases enhance *in vitro* replication and plus-strand RNA synthesis, and the accumulation of TBSV RNA in yeast and plants. We also demonstrate that AtRH2 and AtRH5 helicases work synergistically with the DDX3-like AtRH20 helicase (that binds to the 3′ promoter sequence) to facilitate plus-strand synthesis in an asymmetric manner. Altogether, these co-opted host DEAD box helicases greatly enhance TBSV replication by interacting with the viral (−)RNA and the replication proteins within the VRCs.

## Results

### Overexpression of yeast eIF4AIII-like Fal1p or Dbp3p DEAD-box RNA helicases and the orthologous plant AtRH2 and DDX5-like AtRH5 helicases enhances TBSV RNA replication in yeast and plants

To characterize the functions of host RNA helicases in TBSV replication, first we overexpressed 5 yeast RNA helicases in yeast and tested their effects on TBSV replicon (rep)RNA accumulation via Northern blotting (Fig. S1). These helicases were chosen from the 11 previously identified yeast helicases from several complementary high throughput screens using yeast and tombusviruses [26], [28], [35], [36], [38], [53], [54]. Overexpression of all 5 host RNA helicases increased TBSV accumulation, with the yeast Dbp3p showing the highest (over 2-fold increase) stimulation (Fig. S1A–B). The overexpression of these host helicases did not affect the accumulation of p33 replication protein (Fig. S1A–B), suggesting that the effects of this group of RNA helicases are not through increased translation of viral replication proteins. Altogether, the observed 30-to-140% increase in TBSV RNA accumulation due to overexpression of these yeast helicases is significant since overexpression of most yeast proteins nonspecifically reduces TBSV accumulation by 20–30% as demonstrated before based on individual overexpression of 5,500 yeast proteins [26], [54]. Thus, these helicases likely play stimulatory roles in TBSV replication.

**Figure 1:**
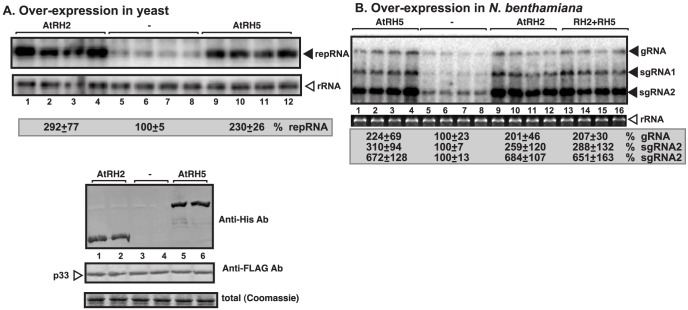
Stimulation of tombusvirus RNA accumulation by over-expression of the eIF4IIIA-like AtRH2 and the DDX5-like AtRH5 DEAD-box helicases in yeast and *N. benthamiana*. (A) Expression of AtRH2 and AtRH5 in yeast (BY4741) enhances TBSV repRNA accumulation. Top panel: Replication of the TBSV repRNA was measured by Northern blotting 14 h after initiation of TBSV replication. The accumulation level of repRNA was normalized based on the ribosomal (r)RNA. Each sample is obtained from different yeast colonies. Middle and bottom panels: The accumulation levels of His_6_-AtRH2 and His_6_-AtRH5 and FLAG-p33 were tested by Western blotting and total protein loading is shown by SDS-PAGE. Each experiment was repeated twice. (B) Expression of AtRH2 and AtRH5 were done separately or together in *N. benthamiana* leaves by agroinfiltration. The same leaves were co-infiltrated with *Agrobacterium* carrying a plasmid to launch *Cucumber necrosis virus* (CNV, a close relative of TBSV) replication from the 35S promoter. The control samples were obtained from leaves expressing no proteins (lanes 5–8). Total RNA was extracted from leaves 2.5 days after agroinfiltration that launched CNV replication. The accumulation of CNV gRNA and subgenomic (sg)RNAs in *N. benthamiana* leaves was measured by Northern blotting (Top panel). The ribosomal RNA (rRNA) was used as a loading control and shown in agarose gel stained with ethidium-bromide (bottom panel).

**Figure 2:**
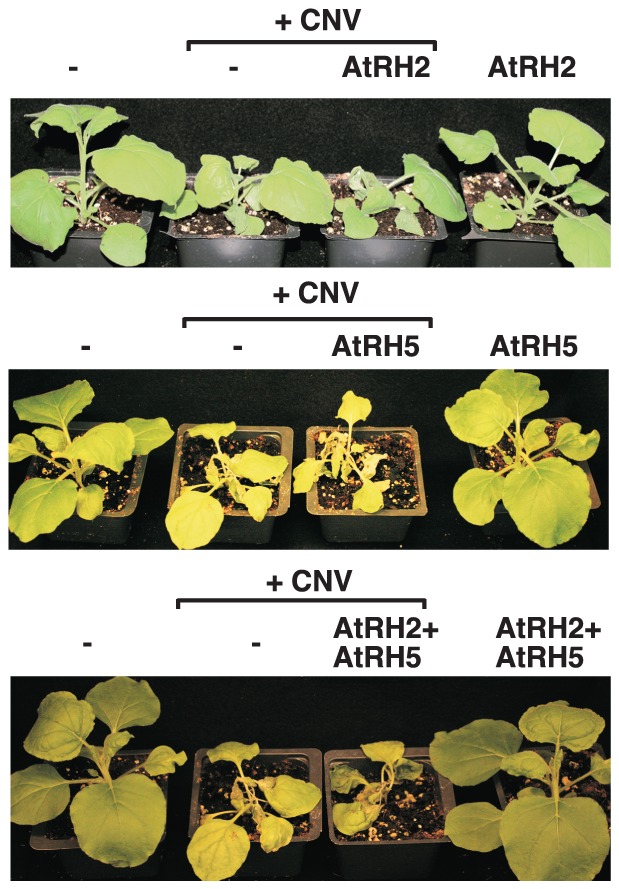
Over-expression of AtRH2 and AtRH5 in *N. benthamiana* accelerates the rapid necrosis caused by systemic CNV infection. The pictures were taken 7 days after agroinfiltration.

For additional in-depth studies, we have selected the yeast Dbp3p and the highly similar Fal1p (human eIF4AIII-like) together with the orthologous *Arabidopsis* AtRH2 (Fal1p ortholog and human eIF4AIII-like) and AtRH5 (Dbp3p ortholog, human DDX5-like) helicases [44]]. Overexpression of AtRH2 and AtRH5 stimulated (up to 3-fold increase) TBSV repRNA accumulation in yeast (Fig. 1A). The stimulation of tombusvirus accumulation (we used *Cucumber necrosis virus*, CNV, which is very closely related to TBSV) was also robust in *Nicotiana benthamiana* host plants when AtRH2 and AtRH5 RNA helicases were overexpressed (up to ∼2-fold increase in tombusvirus genomic RNA accumulation, and ∼6-fold increase in subgenomic RNA2 accumulation, Fig. 1B, lanes 1–4 and 9–12 versus 5–8), suggesting that these cellular helicases are important host factors. While overexpression of AtRH2 and AtRH5 did not affect the phenotype of uninoculated *N. benthamiana* plants, the tombusvirus-induced symptoms were intensified (Fig. 2) and the symptoms appeared faster (not shown), when compared with the control host plants not overexpressing the AtRH2 and AtRH5 helicases. Simultaneous co-overexpression of AtRH2 and AtRH5 increased tombusvirus replication by up to 2-fold (Fig. 1B, lanes 13–16), similar to the level obtained with individual overexpression of AtRH2 and AtRH5. Also, the symptoms induced by tombusvirus infection in plants with co-overexpression of AtRH2 and AtRH5 were comparable to those induced by the individual overexpression of AtRH2 and AtRH5 (Fig. 2). Therefore, it is likely that AtRH2 and AtRH5 play comparable and overlapping functions in tombusvirus replication. Overall, the host helicase overexpression studies in yeast and plant established that AtRH2 and AtRH5 helicases and the orthologous yeast Dbp3p and Fal1p helicases could support increased level of tombusvirus RNA replication in host cells.

### Novel pro-viral function of AtRH2, AtRH5 and the yeast Dbp3p and Fal1p is to bind to the viral replication enhancer present in the tombusvirus minus-strand RNA

**Figure 3:**
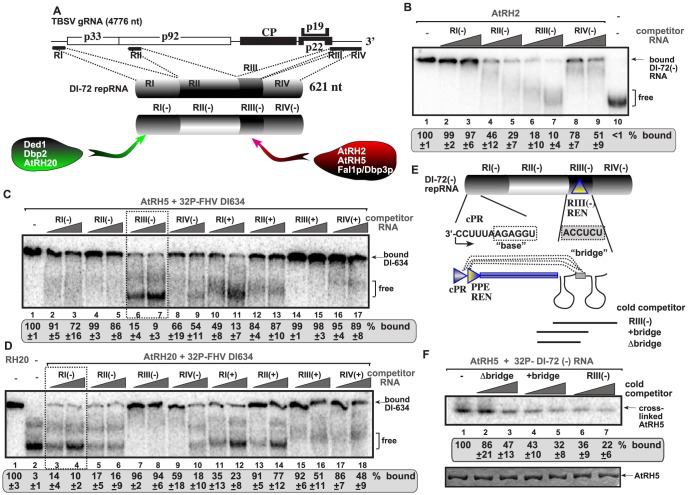
AtRH2 and AtRH5 bind to the RIII(−) replication enhancer element in the TBSV (−)RNA. (A) Schematic representation of the four regions carrying *cis*-acting sequences in the genomic RNA and DI-72 repRNA used in the binding assay. Specific binding by the various cellular DEAD-box helicases are shown. (B) *In vitro* binding assay with purified AtRH2. The assay contained the ^32^P-labeled DI-72 (−)repRNA (∼0.1 pmol) plus increasing amount of unlabeled competitor RNAs, each used in the same amounts, including RI(−) (3 and 6 pmol), RII(−) (2 and 4 pmol), RIII(−) (5 and 10 pmol) or RIV(−) (4 and 8 pmol). The free or AtRH2-bound ssRNA was separated on nondenaturing 5% acrylamide gels. (C) RNA gel shift analysis shows that AtRH5 binds the most efficiently to RIII(−). ^32^P-labeled DI-634 (−)repRNA template (∼0.1 pmol) from FHV and unlabeled competitor RNAs (2 and 4 pmol) representing one of the four regions of TBSV DI-72 RNA from both RNA strands (see panel A) were used in the competition assay. The AtRH5 - ^32^P-labeled ssRNA complex was visualized on nondenaturing 5% acrylamide gels. Each experiment was repeated at least three times. Note that we used the heterologous FHV DI-634 (−)RNA in the binding assay to allow comparison of (+) versus (−)RNA regions of TBSV RNA. The template competition assay showed efficient binding/competition by the RIII(−) and RI(+) sequences for AtRH5. (D) Comparable viral RNA binding assay reveals different binding specificity for DDX3-like AtRH20 and the DDX5-like AtRH5 DEAD-box helicases. See additional details in panel C. The template competition assay showed efficient binding/competition by the RI(−), RII(−), RIV(−) and RI(+) sequences for AtRH20. (E) Schematic representation of the long-range RNA-RNA interaction between the “base” sequence in the cPR promoter in RI(−) and the complementary “bridge” sequence in RIII(−) REN [56]. The competitor RNAs used in panel F are shown schematically. (F) The bridge sequence contributes to binding of RIII(−) to AtRH5 *in vitro*. Top image: RNA-binding analysis of AtRH5 – DI-72 (−)repRNA interaction after UV cross-linking. ^32^P-labeled DI-72 (−)repRNA was used in the absence (lane 1) or presence of various cold competitor RNAs (lanes 2–7) as shown in panel E. Bottom image: SDS-PAGE shows the purified AtRH5 after UV cross-linking to demonstrate comparable sample loading.

To test if AtRH2, AtRH5 and the yeast Dbp3p and Fal1p play a comparable role with the previously analyzed yeast Ded1p (human DDX3-like) and Dbp2p (human p68-like) and the ortologous AtRH20 DEAD-box helicases [30], [49], first we performed *in vitro* RNA binding experiments with affinity-purified recombinant helicase proteins. Using four different *cis*-acting regions present in the TBSV (−)repRNA (Fig. 3A), we found that AtRH2 and AtRH5 bind to a unique *cis*-acting sequence in a 5′ proximal region of the minus-strand RNA, called RIII(−), which carries a well-defined RNA replication enhancer (REN) element (Fig. 3B, lanes 6–7; and S2A, lanes 5–6) [55], [56]. The recombinant yeast Dbp3p and Fal1p RNA helicases showed similar RNA binding characteristics to the RIII(−) REN *in vitro* (Fig. S2B–C).

Importantly, binding of AtRH2, AtRH5 and the yeast Dbp3p and Fal1p to the TBSV RIII(−) REN element is a novel feature for co-opted host helicases. Indeed, the previously characterized AtRH20 and the yeast Ded1p and Dbp2p DEAD-box helicases bound the most efficiently to RI(−) sequence carrying the plus-strand initiation promoter (Fig. 3A; Fig. 3D versus 3C) [30], [49]. This striking difference in recognition of two separate *cis*-acting elements [indeed RIII(−) REN is located close to the 5′end, while RI(−) promoter region is situated at the 3′ end of the viral (−)RNA, Fig. 3A] by these host helicases indicate that their functions and the mechanism of stimulation of TBSV RNA replication must be different.

To confirm binding of AtRH5 to the RIII(−) REN, we also performed UV cross-linking experiments with ^32^P-labeled DI-72(−) RNA and cold competitors (Fig. 3E). The 82 nt complete RIII(−) REN was a better competitor than similar-sized RNA lacking one of the stem-loop structure and a “bridge” sequence that can base-pair with RI(−) sequence via long-range interaction (Fig. 3F, lanes 6–7 versus 2–3) [57]. Our data also support a role for the bridge sequence in binding to AtRH5 (compare lanes 4–5 with 2–3, Fig. 3F).

### AtRH2 and AtRH5 helicases unwind double-stranded RNA structures within the viral replication enhancer region

**Figure 4:**
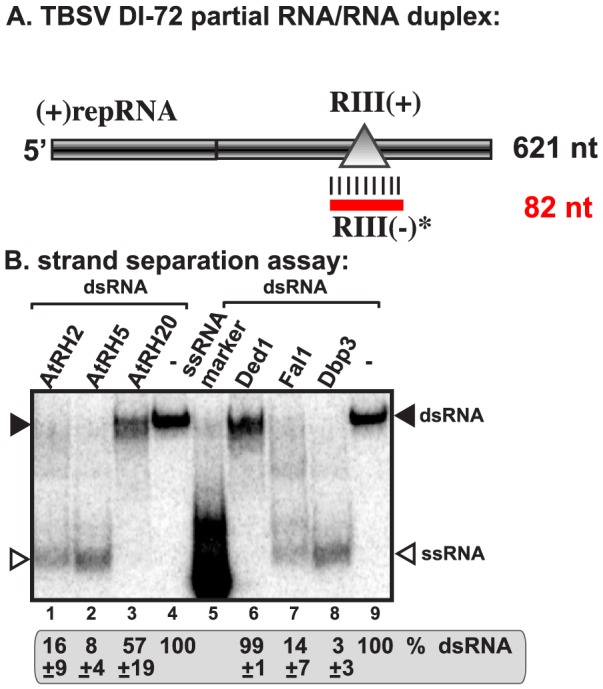
AtRH2 and AtRH5 can unwind short partial RNA/RNA duplex within the RIII(−) REN *in vitro*. (A) Schematic representation of the partial RNA/RNA duplex used in the strand separation assay. The unlabeled template consists of DI-72 (−)repRNA and a complementary RIII(+) RNA, which anneals to the DI-72 (−)repRNA and forms a 82 nt duplex as shown. (B) Representative native gel of ^32^P-labeled RNA products after the *in vitro* strand separation assay. Strand separation assay with a partial RNA/RNA duplex shows the unwinding activity of AtRH2 and AtRH5, Fal1p and Dbp3p (1.0 µg amount) in the presence of ATP. The DDX3-like Ded1p and AtRH20 helicases show only partial unwinding activities under these conditions. Quantification of the RNA/RNA duplex is done with a Phosphorimager.

The efficient binding of AtRH2, AtRH5 and the yeast Dbp3p and Fal1p to the RIII(−) REN region indicates that these helicases might facilitate the unwinding of RNA structures within the RIII(−) REN during replication. Therefore, we tested if recombinant AtRH2 and AtRH5 could unwind partial RNA duplexes, which are known to hinder RdRp-driven RNA synthesis [58], [59]. We chose partial duplex for this assay, because DEAD-box helicases are not processive enzymes and can only unwind short duplexes [40]. Interestingly, addition of purified AtRH2 and AtRH5 unwound the partial RNA duplex (Fig. 4B, lanes 1 and 2) and the yeast Dbp3p and Fal1p showed similar activities (lanes 7–8). In contrast, Ded1p and AtRH20 helicases did not efficiently unwind the RNA duplex formed only within the RIII(−) REN (Fig. 4B, lanes 3 and 6). The failure to unwind this partial duplex by Ded1p and AtRH20 is likely due to the preference of these helicases to bind the RI(−) sequence of the tombusvirus RNA (Fig. 3) [30], [49]. Overall, the unwinding assay further supported that AtRH2, AtRH5 and the yeast Dbp3p and Fal1p have a novel pro-viral function during TBSV replication that is based on interaction with the RIII(−) REN element.

### Replication enhancer-dependent stimulation of *in vitro* TBSV (+)RNA synthesis by AtRH2 and AtRH5 helicases

**Figure 5:**
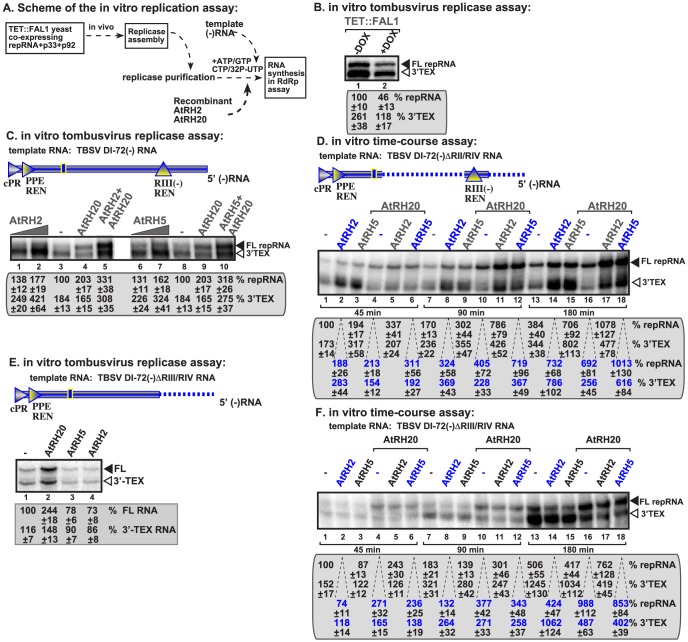
AtRH2 and AtRH5 promote plus-strand synthesis by the affinity-purified tombusvirus replicase. (A) Scheme of the tombusvirus replicase assay. Yeast with depleted eIF4IIIA-like Fal1p co-expressing p33 and p92^pol^ replication proteins and DI-72 (+)repRNA were used to affinity-purify the RNA-free tombusvirus replicase. The *in vitro* assays were programmed with DI-72 (−)repRNA, and they also contained purified recombinant AtRH2, AtRH5 and AtRH20 helicases in addition to ATP/CTP/GTP and ^32^P-UTP. (B) Representative denaturing gel of ^32^P-labeled RNA products synthesized by the purified tombusvirus replicases obtained from yeast either with high Fal1p (−DOX) level or depleted Fal1p (+DOX) is shown. The level of complementary RNA synthesis on DI-72(−) RNA template producing “repRNA” (marked as “FL”, the full-length product, made via *de novo* initiation from the 3′-terminal promoter) was compared in each sample. Note that this replicase preparation also synthesizes 3′-terminal extension products (“3′TEX”). Each experiment was repeated three times. (C) Representative denaturing gel of ^32^P-labeled RNA products synthesized *in vitro* using DI-72(−) template by the purified tombusvirus replicase obtained from yeast with depleted Fal1p in the presence of increasing amounts of purified recombinant AtRH2 (0.2 and 0.4 µg), AtRH5 (0.2 and 0.4 µg) and AtRH20 (1.0 µg) helicases is shown. Samples in lane 5 and 10 contain 0.4 µg AtRH2 and AtRH5, respectively, plus 1.0 µg of AtRH20. Each experiment was repeated three times. (D) Time-course experiment with the purified tombusvirus replicase obtained from yeast with depleted Fal1p using DI-72(−)ΔRII/RIV template. The affinity-purified recombinant AtRH2 (0.4 µg), AtRH5 (0.4 µg) and AtRH20 (0.8 µg) helicases were added to the assay as shown. See further details in panel C. (E) Representative denaturing gel of ^32^P-labeled RNA products synthesized *in vitro* using DI-72(−)ΔRIII/RIV template by the purified tombusvirus replicase obtained from yeast with depleted Fal1p in the presence of purified recombinant AtRH2 (0.4 µg), AtRH5 (0.4 µg) and AtRH20 (1.0 µg) helicases is shown. (F) Time-course experiment with the purified tombusvirus replicase obtained from yeast with depleted Fal1p using DI-72(−)ΔRIII/RIV template. The affinity-purified recombinant AtRH2 (0.4 µg), AtRH5 (0.4 µg) and AtRH20 (0.8 µg) helicases were added to the assay as shown. See further details in panel E.

To test the direct effect of AtRH2 and AtRH5 helicases on TBSV RNA synthesis, we utilized detergent-solubilized and affinity-purified tombusvirus replicase from yeast with down regulated Fal1p (Fig. 5A). The requirement for Fal1p helicase on RNA synthesis was supported by the observed ∼55% decrease of the *in vitro* activity of the purified replicase obtained from yeast with depleted Fal1p when compared with the replicase from yeast expressing Fal1p at high level (Fig. 5B). This purified replicase can only synthesize complementary RNA products on added TBSV templates allowing for the measurement of the level of RNA synthesis [15], [16].

We found that addition of purified recombinant AtRH2 and AtRH5 helicases to the purified tombusvirus replicase (obtained from Fal1p depleted yeast) programmed with the DI-72 (−)repRNA stimulated (+)-strand synthesis by up to 2-fold (Fig. 5C, lanes 2 versus 3 and 7 versus 8). Interestingly, AtRH2 and AtRH5 helicases stimulated the production of both full-length (+)-strand repRNA product (via *de novo* initiation) and the 3′-terminal extension product (3′TEX; due to initiation of complementary RNA synthesis by self-priming from the 3′ end of the template, instead of *de novo* initiation [60]–[62]). This is in contrast with AtRH20 and Ded1p helicases, which stimulated the production of mostly full-length (+)-strand repRNA product (Fig. 5C, lanes 4 and 9) [30], [49]. Time-course experiments with the purified tombusvirus replicase and a minimal template carrying RI(−) and RIII(−) REN sequences confirmed that AtRH2 and AtRH5 helicases stimulated the production of both full-length (+)-strand RNA and 3′TEX products by ∼two-fold at both early and late time points (Fig. 5D).

Interestingly, the stimulation of RNA synthesis by AtRH2 and AtRH5 helicases is lost when we used a (−)repRNA lacking 5′ sequences including RIII(−) REN region (Fig. 5E, lanes 3–4 versus 1). This is in contrast with AtRH20, which was able to stimulate the tombusvirus replicase activity on this template RNA (Fig. 5E, lane 2). This observation was confirmed in *in vitro* time-course experiments with the purified tombusvirus replicase and a template lacking RIII(−) REN by showing the absence of stimulation of RNA synthesis products by AtRH2 and AtRH5 helicases at both early and late time points (Fig. 5F). Therefore, we suggest that AtRH2 and AtRH5 helicases depend on RIII(−) REN to facilitate the overall efficiency of template use and RNA synthesis on the (−)RNA template by the tombusvirus replicase.

**Figure 6:**
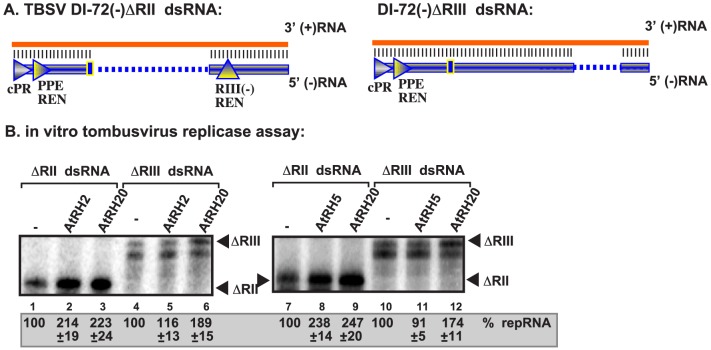
AtRH2 and AtRH5 promote plus-strand synthesis on partial dsRNA templates by the affinity-purified tombusvirus replicase. (A) Scheme of the partial dsRNA templates used. Note the presence or absence of RIII(−) REN in the templates. (B) Representative denaturing gel of ^32^P-labeled RNA products synthesized by the purified tombusvirus replicases obtained from yeast with depleted Fal1p is shown. The level of complementary RNA synthesis on the partial dsRNA templates was compared in the presence of purified recombinant AtRH2 (0.4 µg), AtRH5 (0.4 µg) and AtRH20 (1.0 µg) helicases. Note that this replicase preparation produces mostly (+)RNA products when using dsRNA templates. Each experiment was repeated three times.

To test if AtRH2 and AtRH5 helicases could also stimulate RNA synthesis by the tombusvirus replicase on dsRNA templates, which are formed during TBSV replication (Kovalev et al, in press), we used partial dsRNA duplexes (Fig. 6A). The tombusvirus replicase is inefficient utilizing these dsRNA templates *in vitro* [30], [49], [59]. The addition of recombinant AtRH2 and AtRH5 helicases stimulated RNA synthesis by up to ∼2.5-fold *in vitro* on a partial dsRNA template that had both RI(−) and RIII(−) REN as part of the duplex (Fig. 6A, construct ΔRII and 6B, lanes 2 and 8 versus 1 and 7). This level of stimulation was comparable to that obtained with AtRH20 that targets the RI(−) sequence (Fig. 6B, lanes 3 and 9). AtRH2 and AtRH5 helicases also stimulated RNA synthesis on a complete dsRNA template (Fig. S3A and S3B, lanes 9 and 11 versus 14), producing mostly (+)RNA products (Fig. S3C).

Importantly, the stimulation of RNA synthesis by AtRH2 and AtRH5 helicases was lost when a partial dsRNA template lacking the RIII(−) REN was used (Fig. 6A, construct ΔRIII and 6B, lanes 5 and 11 versus 4 and 10). In contrast, AtRH20 was still able to stimulate RNA synthesis on this template (Fig. 6B, lanes 6 and 12), as predicted based on the ability of AtRH20 to bind to RI(−) sequence [30], [49]. Based on these data, we conclude that AtRH2 and AtRH5 helicases can stimulate (+)RNA synthesis on dsRNA templates in the presence of RIII(−) REN by the tombusvirus replicase.

To further study the roles of AtRH2 and AtRH5 helicases in TBSV replication, we used whole cell extracts (CFE) prepared from yeast containing temperature-sensitive (ts) Fal1p and lacking Dbp3p to support cell-free TBSV replication. TBSV (+)RNA has been shown to perform one full cycle of replication, starting with VRC assembly, (−)RNA synthesis and finally production of excess amount of (+)-strands, in the CFE-based replication assay when purified recombinant p33 and p92^pol^ replication proteins are included [24], [63]. The CFE-based replication assay showed that (+)RNA synthesis decreased by ∼2-fold when compared with the control CFE prepared from yeast with high level of Dbp3p and wt Fal1p (Fig. S4B, lane 4 versus 1). This is in contrast with (−)RNA synthesis (represented by dsRNA product), which was unchanged when the CFE was prepared from dbp3Δ/ts-fal1 yeast versus wt yeast. However, addition of purified AtRH2 or AtRH5 to the CFE assay increased (+)RNA production by ∼60–70%, while the (−)RNA (in the form of dsRNA) was unchanged (Fig. S4C–D). Thus, these data with CFE-based approaches confirm that AtRH2 and AtRH5 and the ortologous yeast helicases are important for (+)RNA synthesis during TBSV replication *in vitro*.

### AtRH2 and AtRH5 are components of the tombusvirus replicase

**Figure 7:**
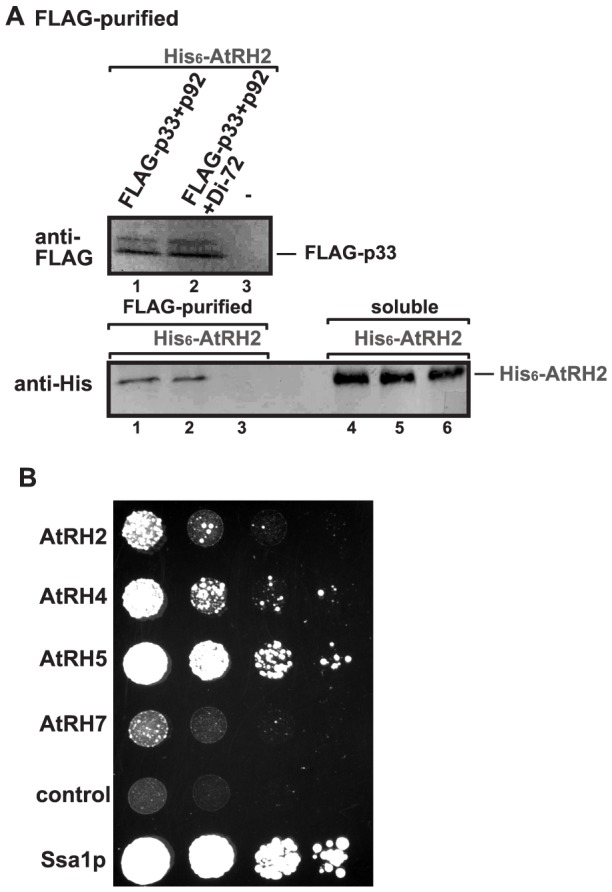
AtRH2 is a component of the tombusvirus replicase in yeast. (A) The membrane-bound tombusvirus replicase was purified via solubilization of the FLAG-tagged p33 and FLAG-p92 from yeast extracts using a FLAG-affinity column (lanes 1–2). Yeast not expressing p33/p92 was used as a control (lane 3). Top panel: Western blot analysis of FLAG-tagged p33 with anti-FLAG antibody. Bottom panel: Western blot analysis of His_6_-tagged AtRH2 with anti-His_6_ antibody in the affinity-purified replicase preparations. Note that “soluble” represents the total protein extract from yeast demonstrating comparable levels of His_6_-AtRH2 in each sample (lanes 4–6). Each experiment was repeated three times. (B) Interaction between *Arabidopsis* DEAD-box helicases and the TBSV p33 replication protein based on the membrane yeast two hybrid assay (split-ubiquitin assay). The bait p33 was co-expressed with the prey full-length host proteins in yeast. The yeast Ssa1p (HSP70 chaperone), and the empty prey vector (NubG) were used as positive and negative controls, respectively. The image shows 10-fold serial dilutions of yeast cultures.

To examine if AtRH2 and AtRH5 helicases are present within the tombusvirus replicase complex, we FLAG affinity-purified the tombusvirus replicase from yeast cells actively replicating TBSV repRNA [15], [28]. The yeast cells also expressed either His_6_-tagged AtRH2 or His_6_-AtRH5 helicases from plasmids. We found that the solubilized and affinity-purified tombusvirus replicase preparation, which is highly active on added templates *in vitro* (not shown), contained His_6_-AtRH2 (Fig. 7A, lane 2), while His_6_-AtRH2 was undetectable in the control yeast sample obtained using the same affinity purification (Fig. 7A, lane 3). Formation of active replicase complex was not necessary for His_6_-AtRH2 to become co-opted since the inactive purified replicase [the tombusvirus replicase is inactive in the absence of the viral RNA; [19], [63]] contained His_6_-AtRH2 when derived from yeast lacking the viral repRNA (Fig. 7A, lane 1). We found that His_6_-AtRH5 showed similar characteristics in these co-purification experiments (Fig. S5A).

To test if the TBSV p33 replication protein interacts directly with AtRH2 and AtRH5, we performed membrane-based split-ubiquitin yeast two-hybrid assay. This assay confirmed the interaction between p33 and AtRH2 and AtRH5 (Fig. 7B). The yeast Dbp3p and Fal1p DEAD-box helicases also interacted with p33 in this assay (Fig. S5B).

To test what region within the TBSV p33 replication protein is involved in the interaction with AtRH2 and AtRH5, we performed pull-down experiments with MBP-tagged p33 derivatives from *E. coli*. These experiments revealed that the RPR-motif in p33 involved in viral RNA-binding was responsible for interacting with both AtRH2 and AtRH5 (Fig. S6A–B). Interestingly, the interaction of p33 with AtRH2 and AtRH5 did not affect the ability of p33 to bind to the viral (+)repRNA *in vitro* (Fig. S7). The interaction between p33 and (+)repRNA is required for recruitment of the viral (+)RNA into replication [17], [64]. Based on the above interaction data, we suggest that the viral p33 replication protein (and p92 replication protein, which within its N-terminal region contains the p33 sequence due to the expression strategy) co-opts AtRH2 and AtRH5 DEAD-box proteins from the host cells into the viral replicase complexes to aid the replication process.

### Synergistic functions of AtRH5 and AtRH20 helicases in promoting tombusvirus replication

**Figure 8:**
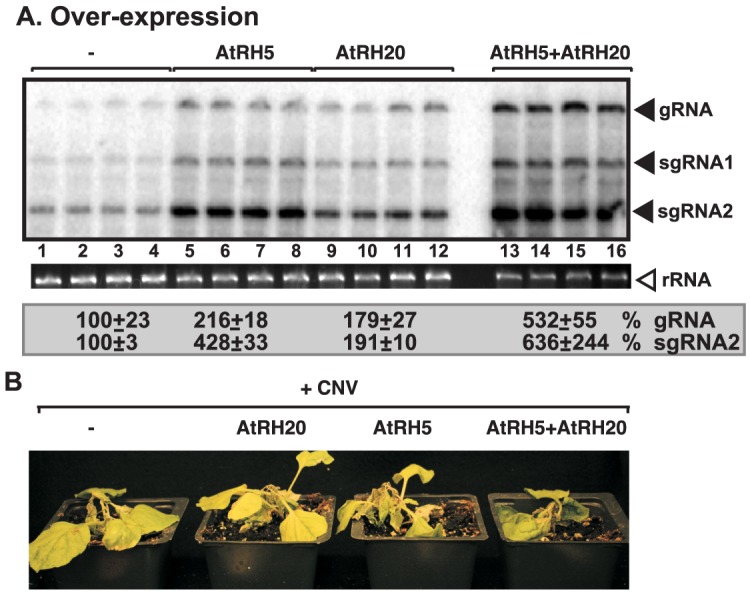
Synergistic stimulatory effect of over-expression of the eIF4IIIA-like AtRH5 and the DDX3-like AtRH20 DEAD-box helicases on tombusvirus RNA accumulation in *N. benthamiana*. (A) Northern blot analysis of the accumulation of CNV gRNA and subgenomic (sg)RNAs in *N. benthamiana* leaves. Expression of AtRH5 and AtRH20 were done separately or together in *N. benthamiana* leaves by agroinfiltration. The same leaves were co-infiltrated with *Agrobacterium* carrying a plasmid to launch CNV replication from the 35S promoter. The control samples were obtained from leaves expressing no proteins (lanes 1–4). Total RNA was extracted from leaves 2.5 days after agroinfiltration after launching CNV replication. The ribosomal RNA (rRNA) was used as a loading control and shown in agarose gel stained with ethidium-bromide (middle panel). (B) Co-over-expression of AtRH5 and AtRH20 in *N. benthamiana* accelerates the rapid necrosis caused by systemic CNV infection. The pictures were taken 12 days after agroinfiltration.

The difference in viral RNA-binding by AtRH2/AtRH5 versus AtRH20 suggests that these groups of helicases could have synergistic effect on tombusvirus replication. This was tested by co-expressing AtRH5 and AtRH20 in *N. benthamiana* leaves replicating the tombusvirus RNA (Fig. 8). Interestingly, AtRH5 and AtRH20 host proteins, when co-expressed together, had the largest (up to ∼5.5-fold, Fig. 8A, lanes 13–16) effect on viral genomic RNA accumulation in comparison with the ∼2-fold increase for separate expression of AtRH20 (lanes 5–8) and AtRH5 (lanes 9–12). Also, the symptom development of tombusvirus-infected plants was the most severe and the fastest when the two helicases were co-expressed (Fig. 8C). Based on these data, we suggest that AtRH5 and AtRH20 have a synergistic effect on tombusvirus replication (see further explanation in discussion).

## Discussion

Replication of (+)RNA viruses is performed by viral replicases (VRCs), which are membrane-bound ribonucleic acid-protein complexes (RNP) [4], [12], [65]. The VRCs likely have to remodel viral RNA structures, including the dsRNA formed during replication. In addition, the viral RNA plays multiple roles, such as template for translation and RNA synthesis, and as a VRC assembly platform and the viral RNA also becomes encapsidated during infection [2]–[4], [66]. It is likely that remodeling of the viral RNAs and RNP complexes during the switch from one step to another requires RNA helicases or RNA chaperones. While the larger RNA viruses over 6,000 nt genome-size all code for RNA helicase-like proteins [47], [48], small RNA viruses usually do not code for RNA helicases. However, the small RNA viruses likely co-opt cellular RNA helicases during infections as shown for TBSV [30], [49]. The yeast DDX3-like Ded1p and the orthologous plant AtRH20 helicases are recruited for TBSV replication to promote (+)-strand RNA synthesis by aiding initiation by the viral RdRp. However, genome-wide screens and global proteomics approaches with TBSV have identified 11 host helicases, suggesting that several host helicases might be co-opted during TBSV infections [28], [34].

In the current work, we have discovered another class of cellular DEAD box RNA helicases, including the yeast eIF4AIII-like Fal1p and Dbp3p and the orthologous plant AtRH2 and DDX5-like AtRH5 DEAD box helicases, which are co-opted by tombusviruses for distinct pro-viral functions. eIF4AIII-like helicases, which assist ribosome biogenesis, are likely involved in local remodeling of large ribosomal RNP structures [52], [67]. Although eIF4AIII helicase has striking homology with eIF4A, yet eIF4AIII is functionally distinct from eIF4A, having no known role in translation initiation, and no interaction with ribosome *in vitro* [52]. The similar Dbp3p helicase, which is also involved in ribosome biogenesis, affects the endonuclease RNase MRP-driven cleavage of pre-ribosomal RNA [51]. It is currently not known how eIF4AIII or Dbp3p RNA helicases are selected for their cellular functions.

In spite of their different cellular functions, Fal1p and Dbp3p helicases play comparable roles during TBSV replication. We find that, unlike the previously characterized AtRH20/Ded1p helicases, the eIF4AIII-like RNA helicases (i.e., AtRH2, AtRH5, Fal1p and Dbp3p) bind to a different *cis*-acting element, the RIII(−) REN, which is present at the 5′ region of the TBSV (−)RNA (Fig. 3A). Also dissimilar with AtRH20/Ded1p helicases is the ability of AtRH2, AtRH5 and the yeast Dbp3p and Fal1p to unwind the dsRNA structure within the RIII(−) REN (Fig. 4). This unique characteristic allows these eIF4AIII-like RNA helicases to perform unique functions that involves the RIII(−) REN.

The AtRH2 and AtRH5 helicases are components of the tombusvirus VRCs as demonstrated by co-purification experiments (Fig. 7 and S5). In addition to binding to the viral RNAs, these eIF4AIII-like RNA helicases also bind to the p33/p92 replication proteins, likely facilitating the recruitment of these cellular helicases into VRCs. It is possible that additional members of the large helicase family could perform a similar function to eIF4AIII-like RNA helicases during TBSV replication. Indeed, 39 RNA helicases are present in yeast and 110–160 RNA helicases are described in plants [44], [68], indicating that additional members of this large protein family could also be involved in TBSV replication.

### A new role for eIF4AIII helicases in viral asymmetrical RNA synthesis

One of the hallmark features of (+)RNA virus replication is the asymmetric nature of RNA synthesis [12], [69]–[71]. The replication process leads to the production of abundant (+)RNA progeny, while the (−)RNA templates are likely sequestered in dsRNA forms within the VRCs. The presented *in vitro* data based on the solubilized/purified tombusvirus replicase and the CFE assay containing the membrane-bound VRC indicate that the eIF4AIII-like RNA helicases can mainly stimulate TBSV (+)-strand synthesis, while their effects on (−)RNA synthesis have not been observed (not shown).

The recombinant eIF4AIII-like RNA helicases enhanced (+)-strand synthesis by the purified recombinant tombusvirus replicase, it is possible that these helicases directly affect TBSV RNA synthesis via affecting the structure of the RNA templates, including the RIII(−)REN. However, we cannot fully exclude that AtRH2, AtRH5 and the yeast Fal1p and Dbp3p helicases could also affect the activity of the VRC due to their interactions with p33 and p92 (Fig. 7). Overall, the recruitment of eIF4AIII-like DEAD-box helicases for replication of a small RNA virus is remarkable, and we suggest that small (+)RNA viruses likely co-opt two or more different host helicases that interact with different *cis*-acting elements in the viral RNA to aid viral replication.

### Model on the synergistic functions of co-opted host helicases in TBSV RNA replication

**Figure 9:**
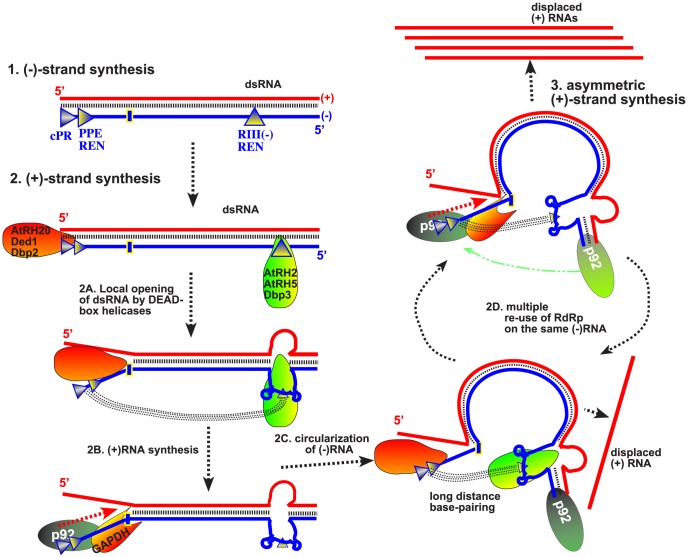
Model on the roles of co-opted cellular helicases in asymmetrical replication of tombusviruses. *Cis*-acting replication elements present at the 3′ and 5′ ends of the viral (−)RNA are recognized by cellular helicases to locally open the viral dsRNA replication form. Namely, the eIF4AIII-like helicases unwind the dsRNA structure within the RIII(−) REN, while the DDX3-like helicases open up the dsRNA structure within RI(−) (as shown schematically). Long-range RNA-RNA interaction between the “bridge” and the cPR region (promoter sequence at the 3′ end) in the (−)RNA is proposed to lead to circularization of the viral (−)RNA, which is still part of the dsRNA. This RNA structure is suggested to facilitate the transfer of the viral p92^pol^ from the 5′ end of the (−)RNA [after completion of the (+)RNA strand synthesis in the previous round] back to the 3′ cPR in the (−)RNA for a new round of (+)RNA strand synthesis. Another co-opted cellular protein, GAPDH (called Tdh2/3 in yeast) is likely involved in this process by facilitating the binding of p92^pol^ to the 3′end of the (−)RNA. Altogether, the re-use of the viral p92^pol^ multiple times on the same (−)RNA template could result in production of multiple (+)RNA progeny, resulting in the characteristic asymmetric viral RNA replication. Note that it is also possible that p92^pol^ is standing still, while the RNA template is moved during RNA synthesis within the membrane-bound VRCs.

Based on their RNA binding features and their abilities to unwind dsRNA regions only locally, we propose that the helicase functions of AtRH2, AtRH5 and the yeast Fal1p and Dbp3p are likely important for unwinding of the RIII(−) REN region in the dsRNA structure formed within the VRCs during TBSV replication. Why is local unwinding of dsRNA within the RIII(−) REN stimulatory for replication? We suggest that the locally opened RIII in the dsRNA form might allow the bridge sequence within the RIII(−) REN to participate in a long-range base-pairing with the 3′end of the (−)RNA, thus bringing the 5′ and 3′ terminal sequences of the (−)RNA in close vicinity (Fig. 9). This could facilitate (+)-strand synthesis and the reutilization of the viral replicases (VRCs) for multiple rounds (as discussed below).

However, the long-distance base pairing between the “bridge” in RIII(−) REN and the cPR promoter in RI(−), both of which are buried in the dsRNA structure, should also depend on opening the dsRNA form within RI(−). This function is unlikely performed by eIF4AIII-like RNA helicases. Instead, we have previously demonstrated that the subverted DDX3-like AtRH20/Ded1p helicases could open up the dsRNA structure within the RI(−) sequence [30].

In summary, based on this and previous publications [21], [30], [49], the emerging picture with TBSV is that this virus utilizes co-opted RNA-binding host proteins to regulate asymmetric viral RNA replication. The recruited host proteins are needed for specific interactions with various *cis*-acting sequences in the viral (−)RNA because the viral p33/p92 replication proteins bind to TBSV (−)RNA nonspecifically [72]. We propose that, first, the recruited eIF4AIII-like RNA helicase proteins bind to RIII(−) REN, while the DDX3-like AtRH20/Ded1p helicases bind to RI(−) sequence. The interactions of two groups of helicases with the viral dsRNA likely opens up the 5′ proximal RIII(−) REN and the 3′ terminal promoter region from the dsRNA structure present in the VRCs. Then, long-distance RNA-RNA interaction between the bridge sequence in the RIII(−) REN and the 3′ terminal sequence [57] could “circularize” the (−)RNA template and bring the p92 RdRp protein from the 5′ end back to the 3′ end for a new round of (+)-strand synthesis (Fig. 9). As proposed earlier [30], [49], an additional function of AtRH20/Ded1p is to further unwind local secondary structure within RI(−) to promote the association of the cellular GAPDH with an AU-rich internal site and proper positioning of the GAPDH-p92 RdRp complex [73] over the (+)-strand initiation promoter, leading to robust (+)RNA synthesis. Therefore, we propose that the synergistic effect between the two groups of subverted helicases, host GAPDH and the viral p92^pol^ might promote efficient recycling of the viral RdRp, resulting in multiple rounds of (+)RNA synthesis on the same dsRNA template (Fig. 9). This strategy could be beneficial for the virus by allowing asymmetric RNA synthesis on dsRNA templates, thus leading to excess amount of progeny (+)RNAs.

It is currently not known if other viruses might also use two different groups of cellular helicases to aid their replication. However, HIV retrovirus, which also lacks viral-coded helicases, has been shown to recruit several cellular helicases, including DDX3, for various steps of its infection cycle [74]–[76]. In addition, host DEAD-box helicases have been shown to affect virus infections, including translation of viral proteins [77]–[79]; viral RNA replication [43], [80]–[83]; subgenomic RNA synthesis [84]; reverse transcription [85]; virus assembly [86]; virus-mediated regulation of host gene transcription [87], and the activity of many anti-viral proteins [88]–[90]. Therefore, the emerging picture is that RNA viruses subvert multiple members of the cellular RNA helicase family during infections.

## Materials and Methods

### Yeast strains and expression plasmids

*Saccharomyces cerevisiae* strain BY4741, Δdbp3 (YKO library) and TET::Fal1 yeast strain (yTHC library), were obtained from Open Biosystems (Huntsville, AL, USA). Ts-Fal1 yeast strain was from a yeast ts strain collection [91]. Yeast strain NMY51 was obtained from Dualsystems. Δdbp3/ts-Fal1 yeast strain was generated as follows: plasmid pYM-14 (EUROSCARF) [92] was used for PCR with primers #5011 and #5012 (Table S1) to amplify the Dbp3 deletion cassette. Ts-Fal1 yeast was transformed with the obtained PCR product and the suitable yeast strain was selected on G418 containing plates. Then, yeast strains were grown in liquid media and genomic DNA was isolated. The correct deletion site was checked by PCR with primers #2215 and #5019 using genomic DNA as a template.

PCR products of yeast helicase genes were obtained as follows: yeast genomic DNA was used as a template for amplification by PCR with primers #4612 and #4613 for *DBP3*; #4569 and #4570 for *DBP5*; #4611 and #4756 for *DBP7*; #2351 and 4825 for *TIF1*; and #4893 and #4894 for *FAL1*. The generated PCR products were digested with *BamH*I and *Xba*I in the case of *DBP3* and *DBP5* and with *Bgl*II and *Xba*I in the case of *DBP7*. Plasmids pYC-His (provided by Dr. Daniel Barajas) and pMalc-2x (New England Biolabs) were digested with *BamH*I and *Xba*I and pPr-N (Dualsystems) was digested with *BamH*I and *Nhe*I and ligated with the similarly treated PCR products of *DBP3, DBP5* and *DBP7*.

The PCR products of plant helicases were obtained as follows: Total RNA was isolated from *A. thaliana* and used for RT-PCR with primers #4816 and #4817 for AtRH2; #4813 and #4871 for AtRH4; #4819 and #4820 for AtRH5; and #4822 and #4823 for AtRH7. The obtained PCR products were digested with *BamH*I and *Sal*I in the case of AtRH2, AtRH4, AtRH7, *FAL*1 and *TIF1* and *Bgl*II and *Sal*I in the case of AtRH5. Plasmid pYC-His was digested with *BamH*I and *Xho*I and plasmids pMalc-2x, pPr-N, pET-30c(+) (for AtRH2 and AtRH5), and pGD-35S (for AtRH2, AtRH4, AtRH5 and AtRH7) were digested with *BamH*I and *Sal*I and ligated to similarly treated PCR products of AtRH2, AtRH4, AtRH5 and AtRH7, *FAL1* and *TIF1*.

Overexpression plasmid pGD-RH20 was obtained as follows: AtRH20 sequence was amplified using primers #4318 and #4473 and pMAL-RH20 [49] as a template. The obtained PCR product was digested with *BamH*I and *Spe*I and inserted into pGD-35S plasmid, which was digested with *BamH*I and *Xba*I. The plasmids pGBK-HIS-Cup-Flag33/Gal-DI-72 expressing Flag-tagged p33 of cucumber necrosis virus (CNV) and the TBSV DI-72 repRNA [93], pGAD-Cup-Flag92 [94], pGD-CNV and pGD-p19 [95] were described earlier.

### Overexpression in plants

Cultures of *Agrobacterium tumefaciens* C58C1 strain carrying pGD-RH2, pGD-RH5, pGD-RH20 (individually) with pGD-CNV and pGD-p19 were prepared and infiltrated into leaves of *N. benthamiana* as described earlier [95]. *Agrobacterium* culture carrying empty pGD-35S plasmid was used as a negative control. During multiple overexpression, we used the *Agrobacterium* cultures with the following density: 0.15 OD_600_ for pGD-CNV, 0.15 for pGD-p19 and 0.7 for one of pGD-RHx (or empty pGD) or 0.35 for each of pGD-RHx when combination of two was applied to the same leaf.

Plant samples from infiltrated leaves were taken 60 hours after infection. RNA was isolated and Northern blot analysis was performed using previously described [14], [95]. For selected samples, proteins were isolated and total proteins level was adjusted based on Coomassie-blue staining. For Western blot analysis, anti-p33 antibody was used (a generous gift of Herman Scholthof, Texas AM University). Pictures of infected plants were taken 7 days after agroinfiltration.

### Recombinant protein purification from *E. coli* and *in vitro* pull-down assay

Recombinant MBP-tagged helicase proteins, the MBP-tagged TBSV p33 and p92 replication proteins and several truncated MBP-tagged p33C derivatives (described earlier) were expressed in *E. coli* and purified as published earlier with modifications [72], [96]. Briefly, the expression plasmids were transformed into *E. coli* strain BL21 (DE3) CodonPlus. Protein expression of the selected helicase proteins was induced by isopropyl-β-D-thiogalactopyranoside (IPTG) for 8 h at 23°C and in the case of viral proteins p33 and p92 at 16°C. After the collection of cells by centrifugation (5,000 rpm for 5 min), the cells were resuspended and sonicated in low-salt column buffer (30 mM HEPES-KOH pH 7.4, 25 mM NaCl, 1 mM EDTA, 10 mM β-mercaptoethanol). To remove cells debris, the lysate was centrifuged at 14,000 rpm for 5 min, followed by supernatant incubation with amylose resin (NEB) for 15 min at 4°C. After careful washing of the columns, the proteins were eluted with MBP-elution buffer [column buffer containing 0.18% (W/V) maltose]. Purification of His_6_-tagged AtRH2 and His_6_-AtRH5 (using plasmids pET30-RH2 or pET30-RH5) was carried out using ProBond (Invitrogen) resin (washed with column buffer, containing 60 mM Imidazole and eluted with column buffer [lacking β-mercaptoethanol], containing 1 M Imidazole), following otherwise the same protocol as for the MBP-tagged proteins. Purified proteins were aliquoted and stored at −80°C. Proteins used for the replication assays were at least 95% pure, as determined by SDS-PAGE (not shown).

For in vitro pull-down assay, purified His_6_-tagged helicase proteins (200 µg) were loaded onto MBP columns, containing bound MBP-tagged p33C derivatives and incubated with mixing for 25 min at 4°C [30]. The columns were washed three times with cold column buffer and the bound protein complexes were eluted with MBP-elution buffer. The eluates were analyzed for the presence of His_6_-tagged proteins by SDS-PAGE, followed by Coomassie blue staining or Western blotting with an anti-His antibody.

### RNA transcripts for in vitro assays

PCR products for “+bridge” and “Δbridge” constructs (Fig. 3E) were prepared as follows: pGBK-HIS-Cup-Flag33/Gal-DI-72 was used as a template for PCR with primer pairs #5480 and #5481, or #5480 and #5482, respectively. The generated PCR products were used to obtain +bridge RNA (86 nt in length) or Δbridge RNA (73 nt in length), each starting from position 368 in DI-72. The RNA transcripts were synthesized on the PCR templates using T7-based transcription [97]. The RNA transcripts used in CFE-based replication or replicase assays were purified as described earlier [97]. The ^32^P-labeled or unlabeled four separate regions (RI-IV, Fig. 3A) and the full-length DI-72 (+) and (−)RNAs were produced as published [72]. Full-length FHV-derived DI-634 (+) or (−)RNA was produced as described [30]. The amounts of transcripts were quantified by UV spectrophotometer (Beckman).

Partial dsRNA duplexes [(−)R124/(+)DI-72 and ((−)R134/(+)DI-72] for *in vitro* replicase assay were prepared as follows: approximately 2 pmol of ^32^P-labeled (−)R134 or (−)R124 were annealed to unlabeled DI-72(+) RNA in STE buffer (10 mM TRIS, pH 8.0, 1 mM EDTA, and 100 m M NaCl) by slowly cooling down the samples (in 20 µl) from 94°C to 25°C in 30 min.

Complete DI-72 dsRNA duplexes were prepared using Replicator RNAi kit (Finnzymes). Briefly, DI-72 (+)-strand RNA, which was synthesized with T7 transcription, was used as a template for synthesis of DI-72 dsRNA by Phi6 RNA polymerase *in vitro*. Purity of dsRNAs was tested with agarose gel-electrophoresis.

### Gel mobility shift assay (EMSA) and strand separation assay

EMSA was performed as described previously [17], with modifications: the binding assay was done in the presence of 20 mM HEPES (pH 7.4), 50 mM NaCl, 10 mM MgCl_2_, 1 mM DTT, 1 mM EDTA 5% glycerol, 6 U of RNasin and 0.1 mg tRNA in a 10 µl reaction volume. Approximately 0.1 pmol of ^32^P-labeled RNA probes, 0.6 µg of purified recombinant proteins and 0.15 or 0.3 µg of unlabeled RNA were used in template competition assay. For the assay, we used 0.02 µg MBP-p33C, 0.1 pmol of ^32^P-labeled SLR2 RNA (the stem-loop sequence from RII) [17] and MBP-AtRH2 (or MBP-AtRH5), in 0.02, 0.06, 0.2 or 0.6 µg amounts.

Strand separation assay was performed as published [58]. Briefly, about 2 pmol of ^32^P-labeled RIII(−) or RI/II/III(−) were annealed to unlabeled DI-72 (+)RNA in STE buffer by slowly cooling down the samples (in 20 µl) from 94°C to 25°C in 30 min. 0.6 µg of purified recombinant helicase proteins (in MBP elution buffer) or MBP as a negative controls were added separately to the partial dsRNA duplex in the RdRp buffer. 2 mM of ATP was added to the reaction. Reaction mixtures were incubated for 15 min at room temperature and loaded onto 5% nondenaturing polyacrylamide gel as described previously [58].

Some samples were treated with proteinase K after the assay. The incubation with proteinase K lasted for 10 min at 37°C using 0.5 µl of proteinase K from stock of 20 mg/ml (dissolved in 50 mM Tris-HCl pH 8.0, supplemented with 1.5 mM CaCl_2_), followed by loading onto 5% nondenaturing polyacrylamide gel.

### UV-cross-linking assay

The UV-cross-linking assay was performed as described [98]. The 10 µl reaction mixture contained 1 µg purified MBP-tagged AtRH2 or AtRH5 proteins, respectively, 0.5 nM ^32^P-UTP-labeled RNA probe, 10 mM HEPES, pH 7.9, 100 mM KCl, 1 mM MgCl_2_, 10% glycerol, and 1 µg tRNA. Unlabeled RNA transcripts of RIII(−) or “+bridge” and “Δbridge” constructs (all RNAs were comparable in length) were used as competitors in 0.1 to 0.3 µg amounts in the competition assay. After the formation of RNA–protein complexes during incubation of the reaction mixtures at room temperature for ∼15 min, we transferred the reaction mixtures to a 96-well plate on ice. To cross-link the RNA and protein, we irradiated the reaction mixtures on ice at 254 nm wave-length for 10 min using an UV Stratalinker 1800 (Stratagene). Then, we digested the unprotected RNAs by 1 mg/ml RNase A for 15 min at 37°C. Samples were mixed and boiled for 10 min in 1× SDS loading dye. Analysis was performed using SDS-PAGE and phosphorimaging [98].

### Helicase proteins co-purification with the viral replicase

Yeast strain Δdbp3 was transformed with plasmids pGBK-HIS-Cup-Flag33/Gal-DI-72, pGAD-Cup-Flagp92 and pYC-Gal-6×HisRH2 or pYC-Gal-6×HisRH5 to co-express the cellular helicases with the viral replication proteins in yeast cells actively replicating the TBSV repRNA. The transformed yeast strains were selected on SC-ULH^−^ plates and then pre-grown overnight at 29°C in selective media containing 2% glucose [49]. After that yeast strains were pelleted by centrifugation at 2,000 rpm for 3 min, we washed the pellet with SC-ULH^−^ media containing 2% galactose and 50 µM CuSO_4_, yeast were grown for 36 hours in SC-ULH^−^ media containing 2% galactose at 23°C. Pelleted yeasts (about 200 mg) were used for affinity-purification of FLAG-p33 and FLAG-p92 with anti-FLAG M2 agarose as published previously [15], [49]. FLAG-p33 was detected with anti-Flag antibody (1/10,000 dilution) and AP-conjugated anti-mouse antibody (1/10,000). His_6_-AtRH2 or His_6_-AtRH5 proteins were detected with anti-His antibody from mouse (1/10,000 dilution) and AP-conjugated anti-mouse (1/10,000) followed by NBT-BCIP detection [15], [49].

### Analysis of helicase protein – viral protein interactions using the split-ubiquitin assay

The split-ubiquitin assay was based on the Dualmembrane kit3 (Dualsystems). pGAD-BT2-N-His33, expressing the CNV p33 replication protein (bait construct), has been published earlier [26]. Yeast strain NMY51 was co-transformed with pGAD-BT2-N-His33 and one of the prey constructs carrying the cDNA for a given helicase or pPR-N-RE as a negative control or pPR-N-*SSA1* as a positive control [26]. Yeasts were plated onto Trp2^−^/Leu2^−^ synthetic minimal medium plates. After transformed colonies were picked with a loop and re-suspended in water, we streaked them onto TLH^−^ (Trp2^−^/Leu2^−^/His2^−^) plates to test for helicase protein-p33 interactions as described [26].

### Over-expression of cellular helicases in yeast

To study the effect of over-expression of yeast and plant helicase proteins on DI-72 repRNA replication in yeast, we transformed *S. cerevisiae* strain BY4741 with three plasmids: pGBK-HIS-Cup-Flag33/Gal-DI-72, pGAD-Cup-Flag92 and one of the following plasmids: pYC-His-RH2, pYC-His-RH4, pYC-His-RH5, pYC-His-RH7, pYC-His-Dbp3, pYC-His-Dbp5, pYC-His-Dbp7, pYC-His-Fal1, pYC-His-Tif1 (empty pYC plasmid was used as a control). After the selection of transformed yeast cells on SC-ULH^−^ plates, they were pre-grown in SC-ULH^−^ media containing 2% glucose for 24 h at 29°C. Then cells were centrifuged at 2,000 rpm for 3 min, washed with SC-ULH^−^ media containing 2% galactose and resuspended in SC-ULH^−^ media containing 2% galactose and 50 µM CuSO_4_. After growing yeast cells for 14 h at 23°C, they were used for total RNA extraction and Northern blotting and Western blotting as previously published [15].

### Tombusvirus replicase purification from yeast and *in vitro* RdRp assay

After yeast strain TET:Fal1 was transformed with plasmids pGBK-HIS-Cup-Flag33/Gal-DI-72 and pGAD-Cup-Flag92, it was pre-grown in SC-ULH^−^ media containing 2% glucose at 29°C. Then yeast cells were centrifuged at 2,000 rpm for 3 min, washed with SC-ULH^−^ media containing 2% galactose and resuspended in SC-ULH^−^ media containing 2% galactose and 50 µM CuSO_4_ in the presence or absence of 1 mg/ml Doxycycline. After growing for 24 h at 23°C, and yeasts were pelleted and the replicase was purified according to a previously published procedure [25]. Briefly, approximately 200 mg of wet yeast cell pellet were resuspended in TG buffer [50 mM Tris–HCl [pH 7.5], 10% glycerol, 15 mM MgCl_2_, 10 mM KCl, 0.5 M NaCl, and 1% [V/V] yeast protease inhibitor cocktail (Ypic)] and homogenized in FastPrep Homogenizer (MP Biomedicals) by glass beads. After the membrane fraction was solubilized with 1 ml TG buffer containing 1% Triton X-100, 1% [V/V] Ypic, Flag-p33 and Flag-p92 were affinity purified on anti-FLAG M2-agarose affinity resin (Sigma). Replicase complex was eluted with 200 ml elution buffer [50 mM Tris–HCl [pH 7.5], 10% glycerol, 15 mM MgCl_2_, 10 mM KCl, 50 mM NaCl, 0.5% Triton X-100, and 0.15 mg/ml Flag peptide (Sigma)].

In vitro RdRp activity assays with the purified tombusvirus replicase preparations were performed by using DI-72 (−)RNA, RI/II (−)RNA or partial dsRNA [such as (−)RI/II/IV/(+)DI-72 or [(−)RI/III/IV/(+)DI-72] or complete dsRNA templates. RNase ONE digestion to remove single-stranded ^32^P-labeled RNA was performed at 37°C for 30 min in a 1× RNase ONE buffer containing 0.1 µl of RNase ONE (Promega) [49].

### In vitro TBSV replication assay in cell-free yeast extract

We prepared cell-free extract (CFE) from BY4741 or Δdbp3/ts-Fal1 yeast strains as described earlier [24]. The CFE-based TBSV replication assays were performed in 20 µl total volume containing 2 µl of CFE, unlabeled 0.15 µg DI-72 (+)RNA or RI/II/IV (+)RNA transcripts, 200 ng purified MBP-p33, 200 ng purified MBP-p92^pol^ and 200 ng purified MBP-tagged helicase proteins. The assays were performed as published [24], [63]. Fractionation of the assay products was done as follows: after 3 h of incubation at 25°C, reaction mixtures were centrifuged at 21,000× g for 10 min to separate the “soluble” (supernatant) and “membrane” (pellet) fraction. Then the membrane fraction was re-suspended in reaction buffer. Both fractions were then treated as separate samples during phenol/chlorophorm extraction, ethanol precipitation. The samples were dissolved in 1×RNA loading dye and analyzed by PAGE electrophoresis in 5% polyacrylamide gel containing 8 M urea with 0.5× Tris-borate/EDTA buffer as described [24], [63]. For the detection of the ^32^P-labeled dsRNAs generated in the CFE assays, we prepared the RNA samples in 1× RNA loading dye (containing 25% formamide), followed by dividing the samples into two equal fractions; one half was loaded on the gel without heat-treatment, while the other half was heat-treated for RNA denaturation at 85°C for 5 min and analyzed by PAGE [27].

## Supporting Information

Figure S1 Over-expression of selected yeast RNA helicases enhanced TBSV repRNA accumulation in yeast. (A) The wt yeast strain was used for the overexpression experiments. Top panel: Northern blot analysis of TBSV repRNA accumulation in yeast overproducing the His_6_-tagged Dbp3p (DDX5-like), Dbp5p, Dbp7p, Fal1p (eIF4AIII-like) and Tif1p (eIF4A-like) DEAD-box helicases from plasmids. These yeast helicases have been identified in previous high throughput screens with TBSV and yeast host. The TBSV repRNA levels were normalized based on rRNA loading. Bottom panel: Northern blot analysis shows the level of ribosomal RNA loading. (B) Top panel: Detection of the overproduced His_6_-tagged Dbp3p, Dbp5p, Dbp7p, Fal1p and Tif1p DEAD-box helicases by Western blotting using anti-His antibody in yeast. Bottom panel: Detection of Flag-tagged p33 and p92^pol^ by Western blotting using anti-Flag antibody. The total protein level in each sample was analyzed by SDS-PAGE and Coommassie-blue staining. Note that all the helicases expressed in yeast are His_6_-tagged at the N-terminus.
(PDF)

Figure S2 AtRH5, and the yeast Dbp3p and Fal1p helicases bind to the RIII(−) replication enhancer element in the TBSV (−)RNA. (A–C) *In vitro* binding assay with 0.6 µg of purified AtRH5, the yeast Dbp3p and Fal1p helicases. The assay contained the ^32^P-labeled DI-72 (−)repRNA (∼0.1 pmol) plus increasing amount of unlabeled competitor RNAs, including RI(−), RII(−), RIII(−) or RIV(−). The free or helicase-bound ssRNA was separated on nondenaturing 5% acrylamide gels, followed by quantification of the bound RNA by a Phosphorimager. See further details in Fig. 3.
(PDF)

Figure S3 Utilization of full DI-72 RNA/RNA duplex by the tombusvirus replicase is facilitated by cellular helicases *in vitro*. (A) Schematic representation of the 621 bp DI-72 RNA/RNA duplex used in the tombusvirus replication assay. (B) Representative denaturing gel of ^32^P-labeled RNA products synthesized *in vitro* using DI-72 RNA/RNA duplex template by the purified tombusvirus replicase obtained from yeast with depleted Fal1p in the presence of 0.4 µg of purified recombinant cellular helicases (except 1.0 µg in case of AtRH20) is shown. Note that lanes 1–6 show samples from the *in vitro* replication assays with the combination of two cellular helicases [i.e., AtRH20 (1.0 µg) plus the shown helicase), while lanes 7–13 show samples with only a single helicase in the assay. (C) Detection of (+) and (−)-stranded RNA products produced by the purified TBSV replicase on the DI-72 RNA/RNA duplex template *in vitro* replication assay containing cellular AtRH5 and AtRH20 helicases (lane 2 in panel B). The blot contains the same amount of cold (+) and (−)-stranded DI-72 RNA, while the ^32^P-labeled repRNA probes were generated as in panel B. The ratio of (+) and (−)-stranded RNA products was estimated.
(PDF)

Figure S4 Cell-free TBSV replication assay supports a role for Fal1p and Dbp3p helicases in plus-strand synthesis. (A) Scheme of the CFE-based TBSV replication assay. TBSV DI-72 (+)repRNA was added to the whole cell extract (CFE) prepared from either WT yeast or Dbp3p-depleted and ts-Fal1p-inactivated yeast strain expressing p33 and p92^pol^ replication proteins. The membrane and soluble fractions were separated at the end of the replication assay by centrifugation. (B) Detection of single- and double-stranded RNA products produced in the cell-free TBSV replication assays. “T” total, “M” membrane fraction, “S” soluble fraction. Note that the ssRNA present in the “S” fraction represents the released (+)repRNA products from the membrane-bound VRCs. (C) Scheme of the CFE-based TBSV replication assay with purified recombinant cellular helicases. (D) Detection of single- and double-stranded RNA products produced in the cell-free TBSV replication assays by denaturing PAGE analysis of the ^32^P-labeled TBSV repRNA products (See panel C). The assay also contained purified recombinant AtRH2 or AtRH5 (0.15 µg) or MBP (the same molar amount as the helicases) as a control. Odd numbered lanes represent replicase products, which were not heat treated (thus both ssRNA and dsRNA products are present), while the even numbered lanes show the heat-treated replicase products (only ssRNA is present). The % of dsRNA and ssRNA in the samples are shown. Note that, in the nondenatured samples, the dsRNA product represents the annealed (−)RNA and the (+)RNA, while the ssRNA products represents the newly made (+)RNA products. Each experiment was repeated three times.
(PDF)

Figure S5 AtRH5 is a component of the tombusvirus replicase in yeast. (A) The membrane-bound tombusvirus replicase was purified via solubilization of the FLAG-tagged p33F from yeast extracts using a FLAG-affinity column (lanes 1–2). Yeast not expressing p33F was used as a control (lane 3). Top panel: Western blot analysis of FLAG-tagged p33F with anti-FLAG antibody. Bottom panel: Western blot analysis of His_6_-tagged AtRH5 with anti-His_6_ antibody in the affinity-purified replicase preparations. Note that “soluble” represents the total protein extract from yeast demonstrating comparable levels of His_6_-AtRH5 in each sample (lanes 4–6). Each experiment was repeated three times. (B) Interaction between yeast DEAD-box helicases and the TBSV p33 replication protein based on the membrane yeast two hybrid assay (split-ubiquitin assay). The bait p33 was co-expressed with the prey full-length host proteins in yeast. The yeast Ssa1p (HSP70 chaperone), and the empty prey vector (NubG) were used as positive and negative controls, respectively. The image shows 10-fold serial dilutions of yeast cultures.
(PDF)

Figure S6 Interaction between AtRH2 and AtRH5 and the TBSV p33 replication protein. (A) A schematic representation of viral p33 and its derivatives used in the binding assay (each MBP-tagged at the N-terminus). The various domains include: TMD, transmembrane domain; RPR, arginine-proline-rich RNA binding domain; P; phosphorylated serine and threonine; S1 and S2 subdomains involved in p33:p33/p92 interaction. The results of the *in vitro* binding experiments are summarized (“+” or “−“, based on two repeats). (B) *In vitro* pull-down assay of His_6_-tagged AtRH5 (lanes 1–4), His_6_-peptide (lanes 5–8) or His_6_-tagged AtRH2 (lanes 11–14) with MBP-p33 derivatives using amylose resin. Top panel: Western blot analysis with anti-His antibody of His_6_-tagged helicases pulled down with MBP-p33 derivatives. Lane 9 contains purified His_6_-tagged AtRH5 as a standard. Bottom panel: Coomasie stained SDS-PAGE gel, showing quality and quantity of purified MBP-p33 derivatives.
(PDF)

Figure S7 AtRH2 and AtRH5 do not inhibit the binding of p33 replication protein to the TBSV (+)RNA. (A) Scheme of the in vitro TBSV (+)RNA binding assay. (B) *In vitro* EMSA binding assay with purified MBP-p33C [an N-terminally truncated version of p33, which shows selective binding to the viral (+)RNA] in the presence of purified AtRH5 or AtRH2. The ^32^P-labeled RNA template was RII(+)-SL (∼0.1 pmol), which is the p33RE [part of RII(+)], and binds selectively to p33. The assay contained 0.02 µg of purified recombinant MBP-p33C, plus 0.02, 0.06, 0.2 or 0.6 µg of purified recombinant AtRH5 or AtRH2, as shown. The samples in lanes 6 and 13 contained 0.6 µg of purified recombinant AtRH5 or AtRH2 in the absence of p33C.
(PDF)
