# Supplementary figures and images for: The Expanding Functions of Cellular Helicases: The Tombusvirus RNA Replication Enhancer Co-opts the Plant eIF4AIII-Like AtRH2 and the DDX5-Like AtRH5 DEAD-Box RNA Helicases to Promote Viral Asymmetric RNA Replication
Source: PLoS Pathog. 2014 Apr 17;10(4):e1004051. doi: 10.1371/journal.ppat.1004051 (PMC3990711; doi:10.1371/journal.ppat.1004051)

### A. Over-expression in yeast

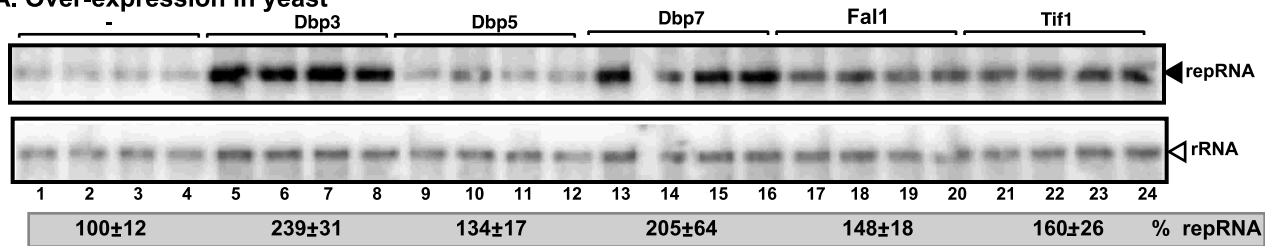

**B**

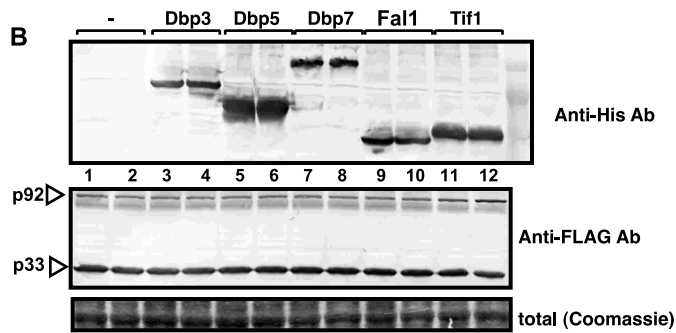

Supplement: Figure S1 — Over-expression of selected yeast RNA helicases enhanced TBSV repRNA accumulation in yeast. (A) The wt yeast strain was used for the overexpression experiments. Top panel: Northern blot analysis of TBSV repRNA accumulation in yeast overproducing the His6-tagged Dbp3p (DDX5-like), Dbp5p, Dbp7p, Fal1p (eIF4AIII-like) and Tif1p (eIF4A-like) DEAD-box helicases from plasmids. These yeast helicases have been identified in previous high throughput screens with TBSV and yeast host. The TBSV repRNA levels were normalized based on rRNA loading. Bottom panel: Northern blot analysis shows the level of ribosomal RNA loading. (B) Top panel: Detection of the overproduced His6-tagged Dbp3p, Dbp5p, Dbp7p, Fal1p and Tif1p DEAD-box helicases by Western blotting using anti-His antibody in yeast. Bottom panel: Detection of Flag-tagged p33 and p92pol by Western blotting using anti-Flag antibody. The total protein level in each sample was analyzed by SDS-PAGE and Coommassie-blue staining. Note that all the helicases expressed in yeast are His6-tagged at the N-terminus. (PDF) [file ppat.1004051.s001.pdf]

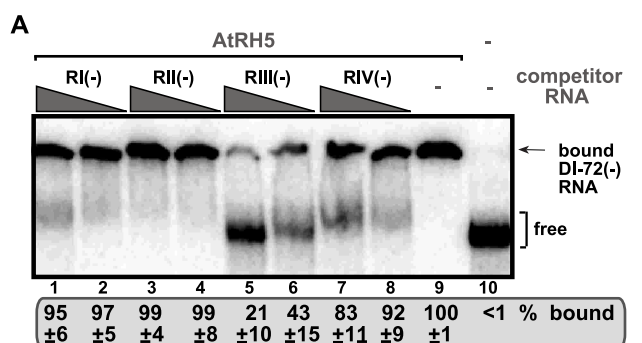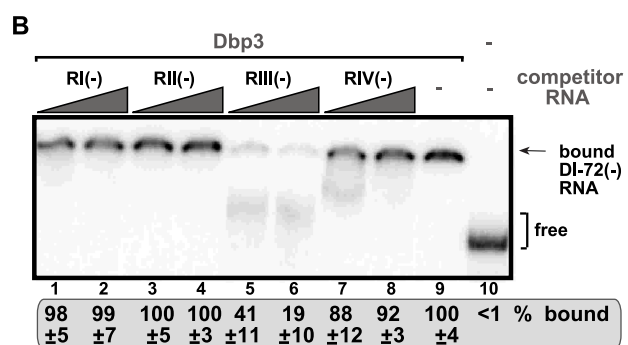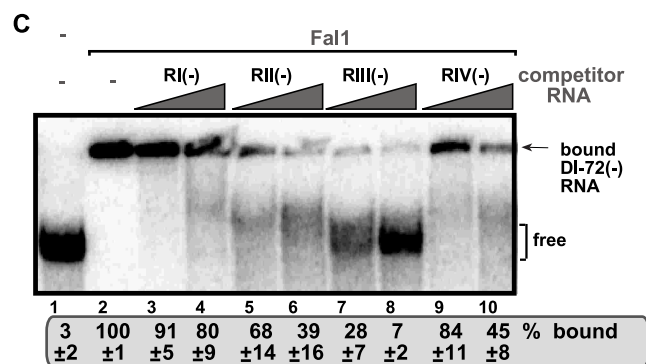

Supplement: Figure S2 — AtRH5, and the yeast Dbp3p and Fal1p helicases bind to the RIII(−) replication enhancer element in the TBSV (−)RNA. (A–C) In vitro binding assay with 0.6 µg of purified AtRH5, the yeast Dbp3p and Fal1p helicases. The assay contained the 32P-labeled DI-72 (−)repRNA (∼0.1 pmol) plus increasing amount of unlabeled competitor RNAs, including RI(−), RII(−), RIII(−) or RIV(−). The free or helicase-bound ssRNA was separated on nondenaturing 5% acrylamide gels, followed by quantification of the bound RNA by a Phosphorimager. See further details in Fig. 3. (PDF) [file ppat.1004051.s002.pdf]

**A. TBSV DI-72 dsRNA:**

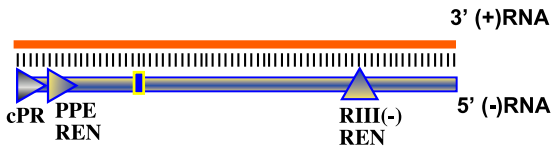

**C**

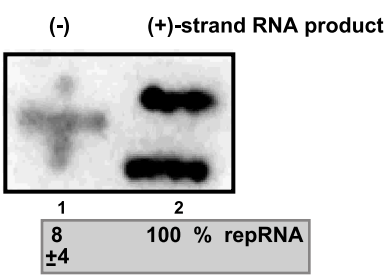

**B. in vitro tombusvirus replicase assay:**

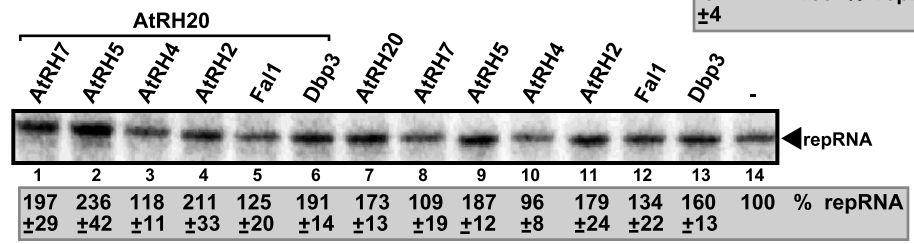

Supplement: Figure S3 — Utilization of full DI-72 RNA/RNA duplex by the tombusvirus replicase is facilitated by cellular helicases in vitro. (A) Schematic representation of the 621 bp DI-72 RNA/RNA duplex used in the tombusvirus replication assay. (B) Representative denaturing gel of 32P-labeled RNA products synthesized in vitro using DI-72 RNA/RNA duplex template by the purified tombusvirus replicase obtained from yeast with depleted Fal1p in the presence of 0.4 µg of purified recombinant cellular helicases (except 1.0 µg in case of AtRH20) is shown. Note that lanes 1–6 show samples from the in vitro replication assays with the combination of two cellular helicases [i.e., AtRH20 (1.0 µg) plus the shown helicase), while lanes 7–13 show samples with only a single helicase in the assay. (C) Detection of (+) and (−)-stranded RNA products produced by the purified TBSV replicase on the DI-72 RNA/RNA duplex template in vitro replication assay containing cellular AtRH5 and AtRH20 helicases (lane 2 in panel B). The blot contains the same amount of cold (+) and (−)-stranded DI-72 RNA, while the 32P-labeled repRNA probes were generated as in panel B. The ratio of (+) and (−)-stranded RNA products was estimated. (PDF) [file ppat.1004051.s003.pdf]

# **A** FLAG-purified

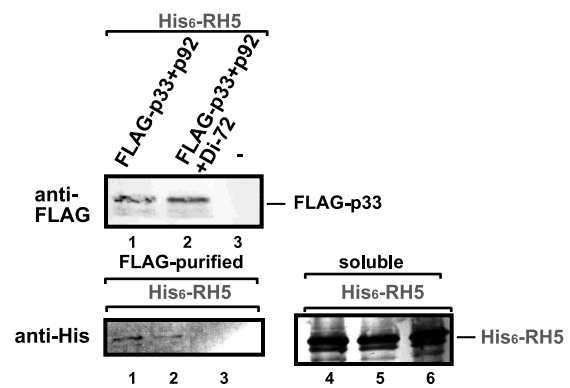

# **B**

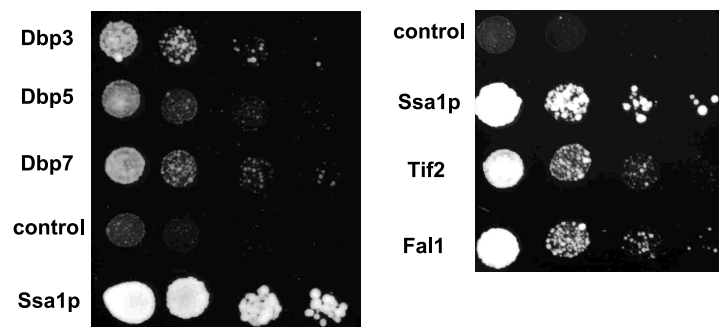

Supplement: Figure S5 — AtRH5 is a component of the tombusvirus replicase in yeast. (A) The membrane-bound tombusvirus replicase was purified via solubilization of the FLAG-tagged p33F from yeast extracts using a FLAG-affinity column (lanes 1–2). Yeast not expressing p33F was used as a control (lane 3). Top panel: Western blot analysis of FLAG-tagged p33F with anti-FLAG antibody. Bottom panel: Western blot analysis of His6-tagged AtRH5 with anti-His6 antibody in the affinity-purified replicase preparations. Note that “soluble” represents the total protein extract from yeast demonstrating comparable levels of His6-AtRH5 in each sample (lanes 4–6). Each experiment was repeated three times. (B) Interaction between yeast DEAD-box helicases and the TBSV p33 replication protein based on the membrane yeast two hybrid assay (split-ubiquitin assay). The bait p33 was co-expressed with the prey full-length host proteins in yeast. The yeast Ssa1p (HSP70 chaperone), and the empty prey vector (NubG) were used as positive and negative controls, respectively. The image shows 10-fold serial dilutions of yeast cultures. (PDF) [file ppat.1004051.s005.pdf]

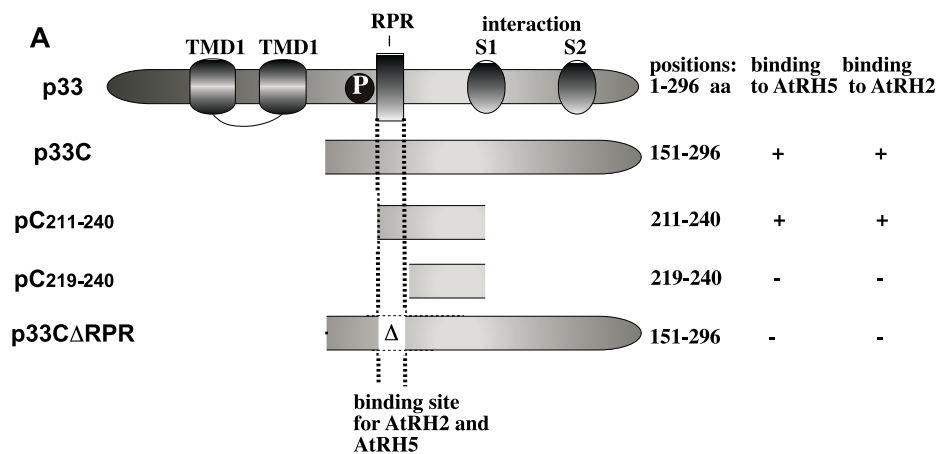

**B Pull-down assay**

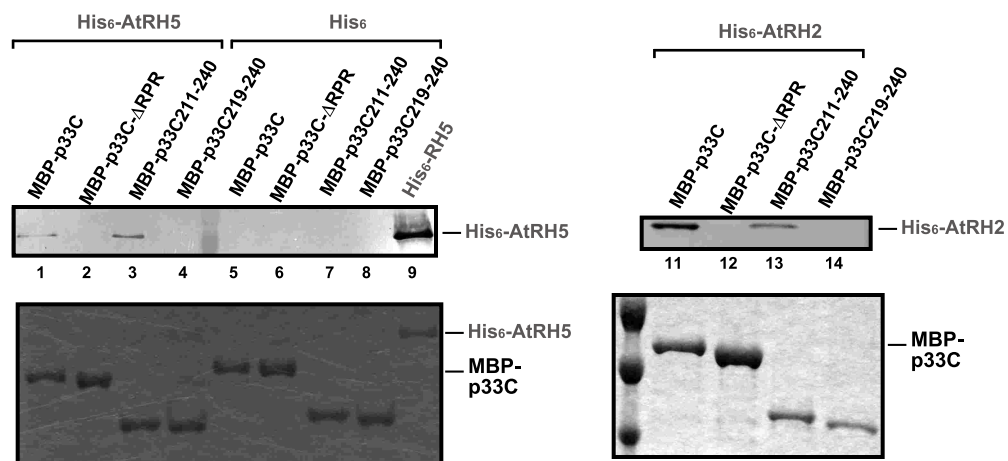

Supplement: Figure S6 — Interaction between AtRH2 and AtRH5 and the TBSV p33 replication protein. (A) A schematic representation of viral p33 and its derivatives used in the binding assay (each MBP-tagged at the N-terminus). The various domains include: TMD, transmembrane domain; RPR, arginine-proline-rich RNA binding domain; P; phosphorylated serine and threonine; S1 and S2 subdomains involved in p33:p33/p92 interaction. The results of the in vitro binding experiments are summarized (“+” or “−“, based on two repeats). (B) In vitro pull-down assay of His6-tagged AtRH5 (lanes 1–4), His6-peptide (lanes 5–8) or His6-tagged AtRH2 (lanes 11–14) with MBP-p33 derivatives using amylose resin. Top panel: Western blot analysis with anti-His antibody of His6-tagged helicases pulled down with MBP-p33 derivatives. Lane 9 contains purified His6-tagged AtRH5 as a standard. Bottom panel: Coomasie stained SDS-PAGE gel, showing quality and quantity of purified MBP-p33 derivatives. (PDF) [file ppat.1004051.s006.pdf]

**A. Scheme of the in vitro assay:**

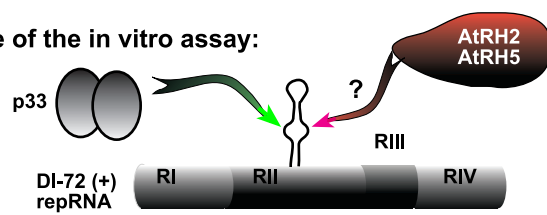

**B. RNA-binding assay:**

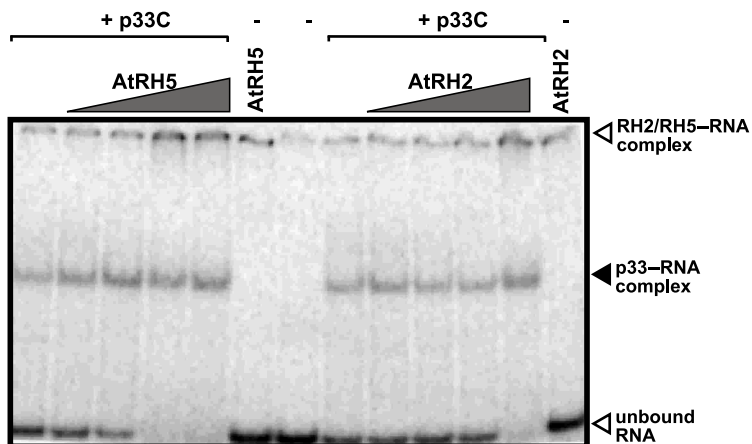

Supplement: Figure S7 — AtRH2 and AtRH5 do not inhibit the binding of p33 replication protein to the TBSV (+)RNA. (A) Scheme of the in vitro TBSV (+)RNA binding assay. (B) In vitro EMSA binding assay with purified MBP-p33C [an N-terminally truncated version of p33, which shows selective binding to the viral (+)RNA] in the presence of purified AtRH5 or AtRH2. The 32P-labeled RNA template was RII(+)-SL (∼0.1 pmol), which is the p33RE [part of RII(+)], and binds selectively to p33. The assay contained 0.02 µg of purified recombinant MBP-p33C, plus 0.02, 0.06, 0.2 or 0.6 µg of purified recombinant AtRH5 or AtRH2, as shown. The samples in lanes 6 and 13 contained 0.6 µg of purified recombinant AtRH5 or AtRH2 in the absence of p33C. (PDF) [file ppat.1004051.s007.pdf]
