# Supplementary material for: The Expanding Functions of Cellular Helicases: The Tombusvirus RNA Replication Enhancer Co-opts the Plant eIF4AIII-Like AtRH2 and the DDX5-Like AtRH5 DEAD-Box RNA Helicases to Promote Viral Asymmetric RNA Replication
Source: PLoS Pathog. 2014 Apr 17;10(4):e1004051. doi: 10.1371/journal.ppat.1004051 (PMC3990711; doi:10.1371/journal.ppat.1004051)
Supplement: Figure S4 — Cell-free TBSV replication assay supports a role for Fal1p and Dbp3p helicases in plus-strand synthesis. (A) Scheme of the CFE-based TBSV replication assay. TBSV DI-72 (+)repRNA was added to the whole cell extract (CFE) prepared from either WT yeast or Dbp3p-depleted and ts-Fal1p-inactivated yeast strain expressing p33 and p92pol replication proteins. The membrane and soluble fractions were separated at the end of the replication assay by centrifugation. (B) Detection of single- and double-stranded RNA products produced in the cell-free TBSV replication assays. “T” total, “M” membrane fraction, “S” soluble fraction. Note that the ssRNA present in the “S” fraction represents the released (+)repRNA products from the membrane-bound VRCs. (C) Scheme of the CFE-based TBSV replication assay with purified recombinant cellular helicases. (D) Detection of single- and double-stranded RNA products produced in the cell-free TBSV replication assays by denaturing PAGE analysis of the 32P-labeled TBSV repRNA products (See panel C). The assay also contained purified recombinant AtRH2 or AtRH5 (0.15 µg) or MBP (the same molar amount as the helicases) as a control. Odd numbered lanes represent replicase products, which were not heat treated (thus both ssRNA and dsRNA products are present), while the even numbered lanes show the heat-treated replicase products (only ssRNA is present). The % of dsRNA and ssRNA in the samples are shown. Note that, in the nondenatured samples, the dsRNA product represents the annealed (−)RNA and the (+)RNA, while the ssRNA products represents the newly made (+)RNA products. Each experiment was repeated three times. (PDF) [file ppat.1004051.s004.pdf]

A. Scheme of the CFE replication assay:

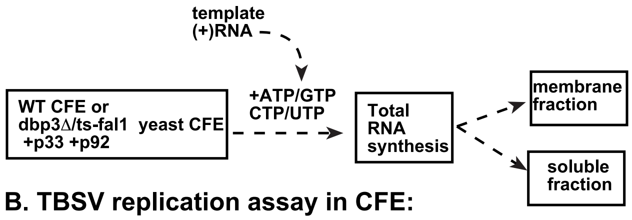

B. TBSV replication assay in CFE:

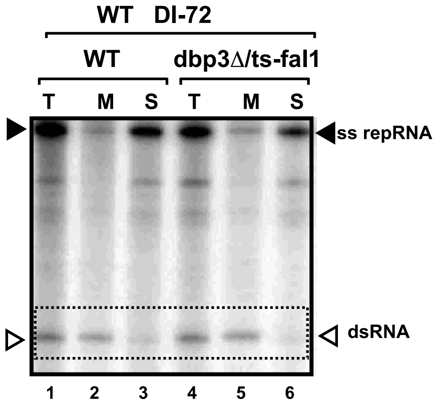

100	10 ±2	45 ±7	57 ±8	12 ±3	28 ±6	% ss repRNA
13	10 ±2	2 ±1	12 ±1	11 ±2	1	% ds repRNA

C. Scheme of the CFE replication assay:

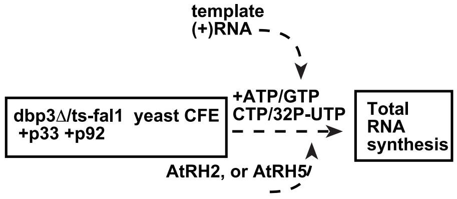

D

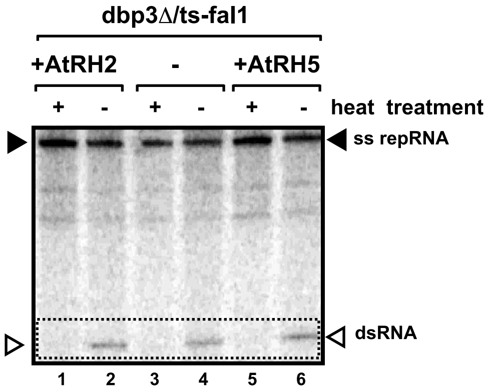

223 ±25	156 ±11	161 ±13	100	214 ±22	167 ±18	% ss repRNA
nd	49 ±8	nd	45 ±7	nd	51 ±8	%dsRNA
